# The Ionospheric Scintillation Effects on the BeiDou Signal Receiver

**DOI:** 10.3390/s16111883

**Published:** 2016-11-09

**Authors:** Zhijun He, Hongbo Zhao, Wenquan Feng

**Affiliations:** School of Electronic and Information Engineering, Beihang University, Xueyuan Road No. 37, Haidian District, Beijing 100191, China; zy1402120@buaa.edu.cn (Z.H.); buaafwq@buaa.edu.cn (W.F.)

**Keywords:** ionospheric scintillation, acquisition process, tracking process, BeiDou system, multiple-phase screen theory

## Abstract

Irregularities in the Earth’s ionosphere can make the amplitude and phase of radio signals fluctuate rapidly, which is known as ionospheric scintillation. Severe ionospheric scintillation could affect the performance of the Global Navigation Satellite System (GNSS). Currently, the Multiple Phase Screen (MPS) technique is widely used in solving problems caused by weak and strong scintillations. Considering that Southern China is mainly located in the area where moderate and intense scintillation occur frequently, this paper built a model based on the MPS technique and discussed the scintillation impacts on China’s BeiDou navigation system. By using the BeiDou B1I signal, this paper analyzed the scintillation effects on the receiver, which includes the acquisition and tracking process. For acquisition process, this paper focused on the correlation peak and acquisition probability. For the tracking process, this paper focused on the carrier tracking loop and the code tracking loop. Simulation results show that under high scintillation intensity, the phase fluctuation could be −1.13 ± 0.087 rad to 1.40 ± 0.087 rad and the relative amplitude fluctuation could be −10 dB to 8 dB. As the scintillation intensity increased, the average correlation peak would decrease more than 8%, which could thus degrade acquisition performance. On the other hand, when the signal-to-noise ratio (SNR) is comparatively lower, the influence of strong scintillation on the phase locked loop (PLL) is much higher than that of weak scintillation. As the scintillation becomes more intense, PLL variance could consequently results in an error of more than 2.02 cm in carrier-phase based ranging. In addition, the delay locked loop (DLL) simulation results indicated that the pseudo-range error caused by strong scintillation could be more than 4 m and the consequent impact on positioning accuracy could be more than 6 m.

## 1. Introduction

The ionosphere is located from 60 km to 1000 km above the Earth and filled with a large number of electrons and plasmas. Irregularities in the ionosphere could cause inhomogeneities in the refractivity of radio frequency (RF) signals while the signals are traveling through the ionosphere, which is known as ionospheric scintillation [1]. Ionospheric scintillation could affect radio signals ranging from 10 MHz to 10 GHz, and the phenomenon varies with time and space. Generally speaking, scintillation is severer in the equatorial area and high-latitude regions than in the middle-latitude area.

The performance of satellite communication could be degraded due to the presence of ionospheric scintillation. Global Navigation Satellite System (GNSS) satellite-receiver links traveling through the atmosphere are also vulnerable to ionospheric scintillation [2]. This could results in some inevitable harmful effects such as signal fading and loss of signal tracking. Signal fading could cause a decline of signal-to-noise ratio (SNR), which could result in degradation of positioning accuracy, and loss of signal tracking could lead to navigation system failure [3]. Moreover, scintillation could also increase the errors of pseudo range and carrier-phase range measurement, and thus have harmful impacts on high precision positioning. Since the low-latitude regions in Southern China are in the equatorial magnetic anomaly area where the scintillation phenomenon is severer and occurs more frequently, it is important to study the its impacts on the BeiDou Navigation Satellite System (BDS).

So far, there have been many studies on ionospheric problem. Zhao used single phase screen model to evaluate the scintillation influence on signal amplitude and phase, and then provided an analysis of how the acquisition process would be affected [4]. Feng and Liu studied the statistical characteristics of amplitude scintillation on the basis of observation data and built an ionospheric scintillation model with a Nakagami-m distribution [5]. Conker and Arini modeled the effects of ionospheric scintillation on Global Position System (GPS)/Satellite-Based Augmentation System (SBAS) and user receiver’s performance, and then developed a receiver model including the effects of scintillation on the tracking process [6]. Béniguel proposed the Global Ionospheric Propagation Model (GISM). GISM used a Multiple Phase Screen (MPS) technique which is based on the resolution of the parabolic equation (PE). They provided the statistical characteristics of the transmitted signals including the scintillation index, the fade durations and so on. By using GISM, Béniguel’s group analyzed the effects of ionospheric scintillation on many aspects, including signal tracking and position accuracy [7,8]. Ho and Abdullah used the GISM to compute amplitude scintillation parameters for GPS satellite signals and calculate the positioning error by the using output data from the model [9]. Zhang and Guo discussed the influence of ionospheric scintillation on Precise Point Positioning (PPP) and analyzed three possible reasons for accuracy degradation caused by scintillations [10]. The Concept for Ionospheric Scintillation Mitigation for Professional GNSS in Latin America (CIGALA) and Countering GNSS high Accuracy applications Limitations due to Ionospheric disturbances in Brazil (CALIBRA) projects, which are funded by the European Commission, developed new algorithms and approaches for high accuracy GNSS techniques to tackle the effects of ionospheric disturbances. The projects built a network database based on eight GNSS stations and tested the approaches in precision agriculture. Vani and Shimabukuro developed a software called Ionospheric Scintillation Monitor Receivers (ISMR) Query Tool, which could generate real-time scintillation reports. Based on ISMR data, such software provided a capability to identify specific behaviors of ionospheric activity through data visualization and data mining [11]. Based on CIGALA and CALIBRA, Veettil and Marcio focused their research on Latin America, especially Brazil. They modeled the ionospheric scintillation and presented a mitigation strategy. By using the data collected at Brazilian stations, they presented their climatology studies and a discussion on receiver tracking performance [12].

Besides, the MPS technique has been widely used in solving problems with random media like the ionosphere. Rino proposed a power law phase screen model for both weak and strong scintillation [13]. Knepp used the MPS technique to analyze the wave propagation through a turbulent ionized medium and compared it with theoretical results for both weak and strong scintillation [14]. Carrano et al. developed the Radio Occultation Scintillation Simulator (ROSS). They used the MPS technique to simulate the forward scatter of radio waves by irregularities in the equatorial ionosphere during radio occultation experiments [15]. Deshpande developed a model called Satellite-beacon Ionospheric-scintillation Global Model of the upper Atmosphere (SIGMA) to simulate GNSS scintillations for high-latitude areas. They simulated the full 3-D propagation of an obliquely incident satellite signal by using the MPS technique and a split-step method. The outputs from their model included the peak to peak (P2P) variations in the power and phase received on the ground, scintillation indices *S*_4_ and $\sigma_{\emptyset }$ [16,17]. Considering that the MPS technique works well in moderate and intense scintillation cases, this paper utilizes it to model the ionospheric scintillation for the BeiDou system. By using the BeiDou B1I signal, this paper analyzes the scintillation effects on acquisition and tracking. For the acquisition process, this paper mainly focuses on the variation of correlation peak and acquisition probability. For the tracking process, this paper calculates the loop error of phase locked loop (PLL) and delay locked loop (DLL) by the integral of phase variation. Then a Nakagami-m fading model is used to calculate the loss of lock probability. Finally, based on the pseudo-code ranging method, this paper gives an example to evaluate the impact of scintillation on positioning performance. 

On the other hand, the BeiDou satellite navigation system is a self-created satellite navigation system developed in China, which covers most of the Asia-Pacific region. Currently the BeiDou system is still not as mature as GPS, thus how to ensure that the system could work effectively and precisely is an important topic.

The paper is organized as followed: Section 2 shows the basic characteristics of ionospheric irregularities; Section 3 is divided into three parts, the first part presents the formulation of MPS model, the second part presents in detail the computational set up of our simulations, and the third part presents the experimental results; Section 4 analyzes the scintillation influence on the acquisition process for a receiver; Section 5 analyzes the influence on the tracking process which in mainly concerned about PLL and DLL, and finally discusses the influence on position accuracy; Section 6 presents the conclusions of the paper.

## 2. Inhomogeneity Characteristics

As the irregularities in ionosphere are regarded as continuous random medium, we need to characterize the dielectric constant *ε* in a statistical method:

(1) $$\varepsilon =<\varepsilon >[1+\varepsilon_{1}(\vec{r},t)]$$

In Equation (1), <·> means the statistical average and <*ε*> is the background mean value of the dielectric constant:

(2) $$<\varepsilon >=\varepsilon_{0}<\varepsilon_{r}>=\varepsilon_{0}(1-\omega_{p0}^{2}/\omega^{2})$$

In Equation (2), *ω_p_*_0_ is the angular frequency of plasma in accord with corresponding background electron density <*N_e_*>, *ω* is the angular frequency of an incident wave, <*ε_r_>* is the dielectric constant relative to the background medium. $\varepsilon_{1}(\vec{r},t$) is the fluctuation of *ε*, which represents the random fluctuation caused by irregularities. Assuming that *ε*_1_ is in isotropic random field, then it can be written as [18]:

(3) $$\varepsilon_{1}=\beta \cdot \xi$$

(4) $$\begin{array}{ccc} \beta =-\frac{\omega_{p0}^{2}}{\omega^{2}-\omega_{p0}^{2}}=-\frac{4\pi r_{e}<N_{e}(z)>}{k^{2}}=-\frac{k_{p}^{2}}{k^{2}}, & & \xi =\frac{\Delta N_{e}(\vec{r})}{<N_{e}(z)>} \end{array}$$

In Equations (3) and (4), *ξ* is a homogeneous random field with a zero mean value and a standard deviation *σ_ξ_*, it reflects the fluctuation of medium; factor *β* contains all probable frequency relations, representing the dispersion characteristics of medium.

The correlation function of *ξ* is [19]:

(5) $$B_{\xi }({\vec{r}}_{1}-{\vec{r}}_{2})=<\xi ({\vec{r}}_{1})\xi ({\vec{r}}_{2})>$$

According to Wiener-Khinchin theorem, the power spectrum density (PSD) of $B_{\xi }\left(\vec{r}\right)$ can be obtained by Fourier transform as follows:

(6) $$\Phi_{\xi }(\vec{\kappa })={\left(2\pi \right)}^{-3}∭B_{\xi }(\vec{r})\text{exp}(-j\vec{\kappa }\cdot \vec{r})d\vec{r}$$

(7) $$B_{\xi }(\vec{r})=∭\Phi_{\xi }(\vec{\kappa })\text{exp}(j\vec{\kappa }\cdot \vec{r})d\vec{\kappa }$$

In Equations (6) and (7), $\vec{\kappa }=\left(\kappa_{x},\kappa_{y},\kappa_{z}\right)$ is the spatial wavenumber. When it comes to one-dimensional calculation, the equation could be equal to:

(8) $$\Phi_{\xi }(\kappa_{x},y,z)=\frac{1}{2\pi }\int B_{\xi }(\vec{r})\text{exp}(-j\kappa_{x}\cdot x)dx$$

Under the condition of isotropy, $B_{\xi }\left(\vec{r}\right)$ is only related to $\left|{\vec{r}}_{1}-{\vec{r}}_{2}\right|$, then Equations (6) and (7) could be simplified as:

(9) $$\Phi_{\xi }(\kappa )=\frac{1}{2\pi^{2}\kappa }\int_{0}^{\infty }B_{\xi }(r)r\;\text{sin}(\kappa r)dr$$

(10) $$B_{\xi }(r)=\frac{4\pi }{r}\int_{0}^{\infty }\Phi_{\xi }(\kappa )\kappa \;\text{sin}(\kappa r)d\kappa$$

Numerous satellite observation experiments show that under the isotropy condition, the 3-dimensional PSD of irregularities has the following character [20]:

(11) $$\Phi_{\xi }(\kappa )\propto \kappa^{-p}$$

In Equation (11), *p* is the power law index. There have been several expressions of irregularity PSD; the expression used in this paper is the Shkarofsky spectrum [21]:

(12) $$\Phi_{\xi }(\kappa )=\frac{\sigma_{\xi }^{2}{\left(\kappa_{0}l_{i}\right)}^{(p-3)/2}{l_{i}}^{3}}{{\left(2\pi \right)}^{3/2}K_{(p-3)/2}(\kappa_{0}l_{i})}{\left(l_{i}\sqrt{\kappa^{2}+{\kappa_{0}}^{2}}\right)}^{-p/2}\cdot K_{p/2}(l_{i}\sqrt{\kappa^{2}+{\kappa_{0}}^{2}})$$

In Equation (12), *l_i_* and *L*_0_ = 2*π*/*κ*_0_ are the inner and outer scale size of the irregularities, *K_v_* is the second type of modified Bessel function. 

**Figure 1:**
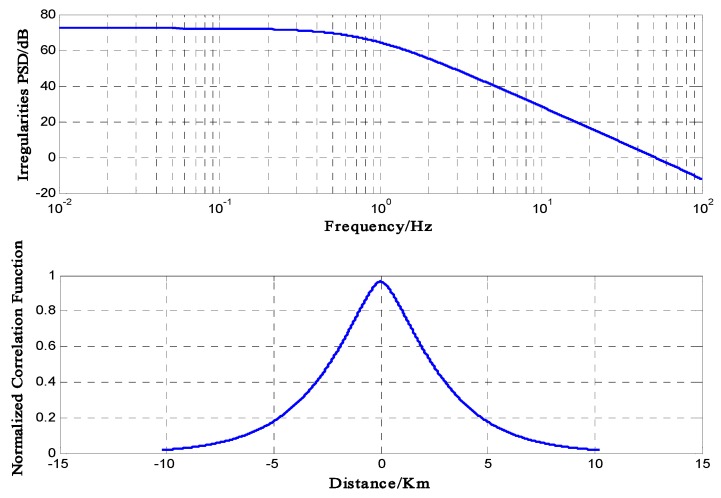
(**top**) PSD and (**bottom**) correlation function of irregularities in the ionosphere.

Figure 1 shows the PSD and correlation function of the ionospheric irregularities for Shkarofsky method with $\vec{\rho }=\left(x,0,0\right)$ and $\sigma_{\xi }^{2}=0.08$.

## 3. Solution of Signal Propagation Based on MPS Technique

### 3.1. Formulation

**Figure 2:**
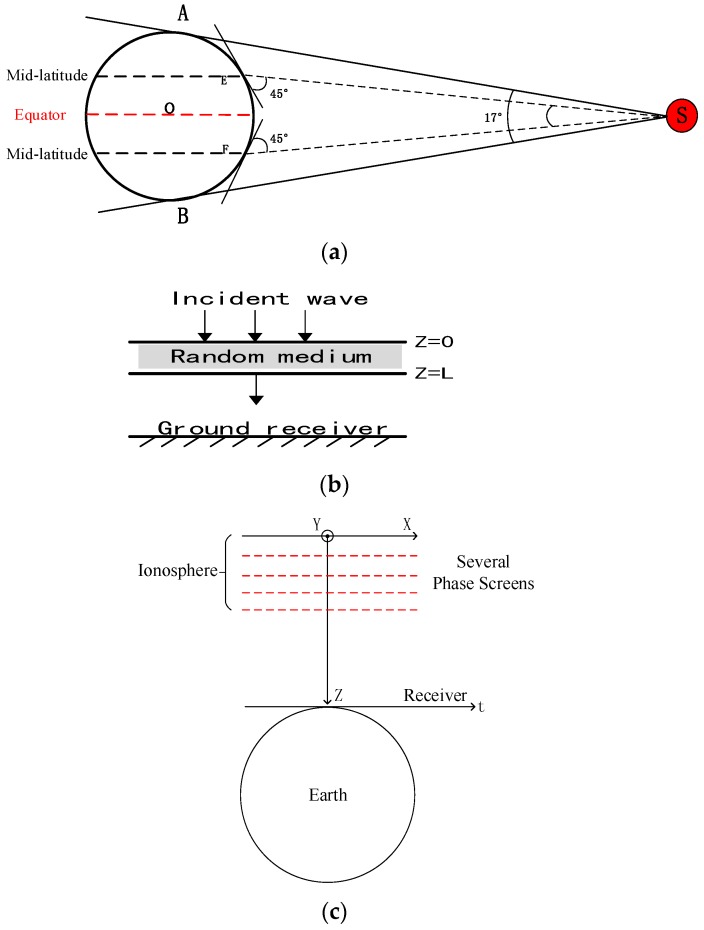
(**a**) Schematic figure of Medium Earth Orbit (GEO); (**b**) Propagation of incident wave; (**c**) Coordinate system.

Ionospheric scintillation is ultimately the fluctuation of phase and amplitude caused by irregularities. Currently, the BeiDou system has ten Medium Earth Orbit (MEO) satellites, five Geostationary Earth Orbit (GEO) satellites and five Inclined Geosynchronous Satellite Orbit (IGSO) satellites. GEO and IGSO are both synchronous satellites. The orbit altitudes of MEO/GEO/IGSO are, 21528 km/35786 km/35786 km, respectively. Generally speaking, MEO and IGSO serve as positioning satellites and GEO communicate with ground stations to exchange telemetry information. This research would particularly focus on the impact of ionospheric scintillation on synchronous satellites. Figure 2a shows a schematic figure.

In Figure 2a, O is the Earth center, S is a satellite, A and B are the tangency points. As shown, ∠ASB=17° indicates the maximum coverage area of a synchronous satellite. Generally, a receiver’s antenna elevation angle would be less than 45° to ensure a high ranging accuracy, so the practical maximum coverage angle ∠ESF would be less than 10°. Thus the maximum angle of the incident wave from a synchronous satellite would be less than 5°. In some cases, for synchronous satellite which is positioned above the equator, the effective coverage area is from equator to mid-latitude as shown in figure.

Based on the reasons mentioned above, this paper make an assumption of vertical incidence situation. As shown in Figure 2b, ionospheric irregularities are assumed to be distributed in the space between layer z=0 and z=L. A time-harmonic electromagnetic wave is incident on the irregular slab at z=0 and received at ground receiver.

In MPS theory, the ionized region is divided into several thin screens with each perpendicular to the direction of propagation and each screen could cause a change on signal phase and amplitude during the propagation. MPS method obeys to Markov approximation [22]: the wave field in any altitude $z^{\prime }$ is only relevant to the irregularities located in $z<z^{\prime }$; propagation distance is much higher than the radial correlation distance. Thus for the MPS method, it is assured to perfectly simulate the deviation of amplitude and phase as long as the thickness and number of phase screen are set small and large enough. Figure 2c shows the established coordinate system.

Let us consider an incident wave with complex amplitude *A*_0_ and after the propagation through a phase screen the wave becomes:

(13) $$u(\vec{\rho },z)=A_{0}\;\text{exp}[\psi (\vec{\rho },z)]=A_{0}\;\text{exp}[\chi (\vec{\rho },z)-jS(\vec{\rho },z)]$$

In Equation (13), $\chi (\vec{\rho },z$) is referred to as the log-amplitude and $S(\vec{\rho },z$) as the phase deviation of the wave. Every time the signal wave pass through a phase screen, $S(\vec{\rho },z$) will increase by $\varphi (\vec{\rho }$):

(14) $$S=S+\varphi (\vec{\rho })$$

According to [14], when the thickness of the screen is set small enough, the irregularities in each phase screen layer are regarded to be distributed uniformly. For a thin phase screen with thickness Δ*z*, the value of $\varphi (\vec{\rho }$) depends on irregularities density $N_{f}(\vec{\rho }$):

(15) $$\varphi (\vec{\rho })=-\lambda r_{e}\int_{0}^{\Delta z}N_{f}(\vec{\rho })<N_{e}>dz$$

In Equation (15), $\vec{\rho }$ is a two-dimensional coordinate vector on the phase screen plane, *λ* is the signal wavelength, <*N_e_*> is the mean value of the electron density in the ionosphere. For ionospheric irregularities, the autocorrelation function of the phase change is related to the autocorrelation function of ionization fluctuations by the following equation [14]:

(16) $$B_{\varphi }(\xi )={N_{e}}^{2}r_{e}^{2}\lambda^{2}\Delta z{\int B}_{Nf}(\xi ,z)dz$$

In Equation (16), *B_Nf_*(*ξ,z*) is the autocorrelation function of the electron density fluctuation. Based on Equations (15) and (16), the PSD of $\varphi (\vec{\rho }$) can be obtained by Fourier transform:

(17) $$\Phi_{\varphi }(\vec{\kappa })=N_{e}^{2}{\left(\lambda r_{e}\right)}^{2}2\pi \Delta z\Phi_{\xi }(\vec{\kappa })$$

**Figure 3:**
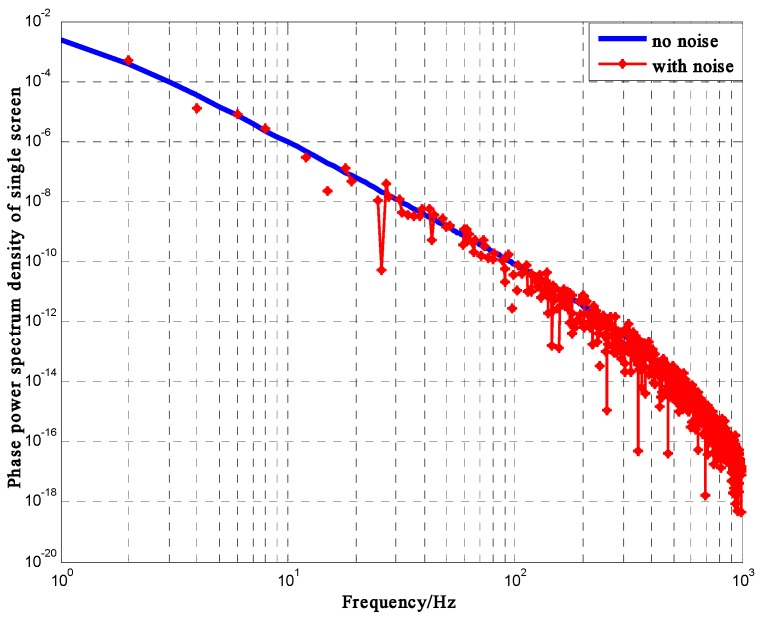
Phase PSD of single phase screen ($\sigma_{\xi }^{2}$ = 0.1, p = 4).

$\Phi_{\xi }\left(\vec{\kappa }\right)$ is shown in Equation (12). Figure 3 shows the PSD of $\varphi (\vec{\rho }$).

The parabolic wave equation is given by [19,23]:

(18) $$-2ik\frac{\partial u}{\partial z}+\nabla^{2}u=-k^{2}\Delta \varepsilon_{1}u(0<z\leq L)$$

In Equation (18), $\nabla^{2}=\frac{\partial^{2}}{\partial x^{2}}+\frac{\partial^{2}}{\partial y^{2}}$ is the transverse Laplace operator. *k* is the wavenumber and $\Delta \varepsilon_{1}$ is the fluctuating part characterizing the random variations caused by irregularities. According to [14], in this case of a vertical incident wave, the irregularities could be assumed infinitely elongated in the *y*-direction, so the equation is limited in two-dimensions with no variable in the *y*-direction. Then the wave propagation can be characterized by the two following equations:

(19) $$\frac{\partial^{2}u}{\partial x^{2}}-2ik\frac{\partial u}{\partial z}+k^{2}\Delta \varepsilon_{1}u=0(z_{n}^{-}<z\leq z_{n}^{+})$$

(20) $$-2jk\frac{\partial u}{\partial z}+\nabla^{2}u=0(z_{n}^{+}<z<z_{n+1}^{-})$$

Equation (19) characterizes the propagation through the *n*-th phase screen and Equation (20) characterizes the propagation in the vacuum space between adjacent screens. Then by using the Kirchhoff’s diffraction formula [24] and Fresnel diffraction theory [24], the recursion formula of log-amplitude and phase deviation from one screen to the next can be obtained:

(21) $$\begin{array}{l} \chi (\vec{\rho })=\frac{k}{2\pi z}\iint S(\vec{\rho^{\prime }})\text{cos}(k{\left|\vec{\rho }-\vec{\rho^{\prime }}\right|}^{2}/2z)\\ +\chi (\vec{\rho^{\prime }})\text{sin}(k{\left|\vec{\rho }-\vec{\rho^{\prime }}\right|}^{2}/2z)+\varphi (\vec{\rho^{\prime }})\text{cos}(k{\left|\vec{\rho }-\vec{\rho^{\prime }}\right|}^{2}/2z)d^{2}\rho^{\prime } \end{array}$$

(22) $$\begin{array}{l} S(\vec{\rho })=\frac{k}{2\pi z}\iint S(\vec{\rho^{\prime }})\text{sin}(k{\left|\vec{\rho }-\vec{\rho^{\prime }}\right|}^{2}/2z)\\ -\chi (\vec{\rho^{\prime }})\text{cos}(k{\left|\vec{\rho }-\vec{\rho^{\prime }}\right|}^{2}/2z)+\varphi (\vec{\rho^{\prime }})\text{sin}(k{\left|\vec{\rho }-\vec{\rho^{\prime }}\right|}^{2}/2z)d^{2}\rho^{\prime } \end{array}$$

In Equations (21) and (22), *z* is the diffraction distance equal to the distance between two adjacent phase screens. The PSD form of the recursion formula is then achieved by Fourier transformation:

(23) $$\begin{array}{l} \Phi_{\chi }(\vec{\kappa })=\frac{1}{2}[\Phi_{S}(\vec{\kappa })+\Phi_{\varphi }(\vec{\kappa })+\Phi_{\chi }(\vec{\kappa })]\\ +\frac{1}{2}[\Phi_{\chi }(\vec{\kappa })-\Phi_{S}(\vec{\kappa })+\Phi_{\varphi }(\vec{\kappa })]\text{cos}(\kappa^{2}z/k+\Phi_{\chi S}(\vec{\kappa })\text{sin}(\kappa^{2}z/k) \end{array}$$

(24) $$\begin{array}{l} \Phi_{S}(\vec{\kappa })=\frac{1}{2}[\Phi_{S}(\vec{\kappa })+\Phi_{\varphi }(\vec{\kappa })+\Phi_{\chi }(\vec{\kappa })]\\ +\frac{1}{2}[\Phi_{S}(\vec{\kappa })+\Phi_{\varphi }(\vec{\kappa })-\Phi_{\chi }(\vec{\kappa })]\text{cos}(\kappa^{2}z/k)-\Phi_{\chi S}(\vec{\kappa })\text{sin}(\kappa^{2}z/k) \end{array}$$

(25) $$\Phi_{\chi S}(\vec{\kappa })=\frac{1}{2}[\Phi_{S}(\vec{\kappa })+\Phi_{\varphi }(\vec{\kappa })-\Phi_{\chi }(\vec{\kappa })]\text{sin}(\kappa^{2}z/k)$$

According to MPS theory, for each single phase screen, the calculation becomes a one-dimensional process due to the infinite distribution of irregularities in *y*-direction, so all the $\vec{\kappa }$ used above should be one-dimensional variables:

(26) $$\vec{\kappa }={\sqrt{\kappa_{x}^{2}+\kappa_{y}^{2}+\kappa_{z}^{2}}|}_{\kappa_{y}=0,\kappa_{z}=0}=\kappa_{x}=\frac{2\pi f}{V_{eff}}$$

In Equation (26), *V_eff_* is the speed at which signals scan the ionospheric phase screen, and $V_{eff}=\frac{V_{s}}{\gamma }$, γ is the ratio of satellite altitude and ionosphere altitude, *V_s_* is the linear velocity of the satellite. For the MPS method, when it comes to the calculation of *V_eff_*, this paper considers the ionosphere as a whole with an altitude of 350 km, which means that for a given satellite, the parameter *V_eff_* in the calculation of each phase screen is regarded as a constant.

By recursion formula, the PSD of log-amplitude and phase deviation in each phase screen can be obtained. Especially, when signal passes through the last screen to ground receiver, *z* should be assigned with the value of the distance between the last screen and ground. After the final step of recursion, the PSD on the ground screen is achieved. To generate the fluctuations of signal phase and amplitude on space field, a sequence of uniformly distributed random numbers is used. The calculation is related to the 1-D discrete Fourier inverse transform of the PSD as described in the following equation:

(27) $$\begin{array}{cc} f(z)=\frac{1}{L_{h}}\sum_{k=0}^{N-1}F(k)\;\text{exp}(j\frac{2\pi k}{N}n) & n=0,1,2\Lambda ,N \end{array}$$

In Equation (27), $L_{h}=\frac{N}{f_{s}}\cdot V_{eff}$ is the length of the phase screen, *N* is the number of sample points, which is equal to grid points on phase screen, *f_s_* is the sample frequency, $\frac{N}{f_{s}}$ is determined by the navigation message. *F*(*k*) is calculated as follows:

(28) $$F(k)=\sqrt{2\pi L_{h}\cdot PSD(\frac{2\pi k}{L_{h}})}\cdot \text{exp}(j\varphi_{k})$$

In Equation (28), *ϕ_k_* is a series of random phase angle with the uniform distribution in (0, 2*π*) and meet the requirement *ϕ_k_* = −*ϕ_−k_*. Considering that the profile is a series of real numbers, the random amplitude and phase fluctuation of the received signal on ground screen can be described as:

(29) $$\chi (n)=\sum_{k=0}^{N-1}\sqrt{\Phi_{\chi }(k\cdot \frac{2\pi }{L_{h}})\cdot \frac{2\pi }{L_{h}}}\cdot \text{cos}(\frac{2\pi nk}{N}+\varphi_{k})$$

(30) $$S(n)=\sum_{k=0}^{N-1}\sqrt{\Phi_{S}(k\cdot \frac{2\pi }{L_{h}})\cdot \frac{2\pi }{L_{h}}}\cdot \text{cos}(\frac{2\pi nk}{N}+\varphi_{k})$$

Generally, ionospheric scintillations are most severe and prevalent at 200 km to 400 km from the ground. To let the distribution of a phase screen adequately represent the actual signal fluctuations, the distribution on the phase screen must contain at least one whole period of signal and the phase-screen length *L_h_* must be at least five times as large as the outer scale size. For the spacing between grid points Δ*q*, it should be set small enough to ensure that the phase change from one grid to the next is less than *π* to satisfy the Nyquist Sampling Theorem. The limitation of *L_h_* and Δ*q* is given by [14]:

(31) $$L_{h}\geq 5L_{0}$$

(32) $$\Delta q\leq \frac{l_{i}}{3}$$

(33) $$\Delta q\leq \pi {\left(-\frac{d^{2}B_{\varphi }(\xi )}{d\xi^{2}}{|}_{\xi =0}\right)}^{-1/2}$$

In Equation (33), *B_ϕ_*(*ξ*) is given in Equation (27). On the other hand, to make sure that the simulation is close enough to the actual propagation, the difference of the function *κ*^2^*z*/*k* between two adjacent values of *κ* must be less than 2*π*. The limitation of *z* is:

(34) $$\frac{\pi^{2}N^{2}z}{k{L_{h}}^{2}}-\frac{\pi^{2}{\left(N-1\right)}^{2}z}{k{L_{h}}^{2}}<2\pi$$

Then the ionospheric scintillation index *S*_4_ can be calculated as follows:

(35) $$S_{4}=\sqrt{\frac{<I^{2}>-{<I>}^{2}}{{<I>}^{2}}}$$

In Equation (35), *I* is the signal intensity, equal to the normalized signal amplitude.

### 3.2. Computational Set up of Simulations

Considering the criteria mentioned above, this paper divides the ionized region into 40 phase screens. For recursion calculation, the input signal amplitude is set to a normalized complex amplitude equal to 1, and the initial PSD of phase and amplitude are set zero. $\frac{N}{f_{s}}=$ 12 s is equal to the time period of two sub-frames of BeiDou navigation message. Some more details of the computational set up of the simulations are shown in Table 1.

### 3.3. Experimental Results

**Figure 4:**
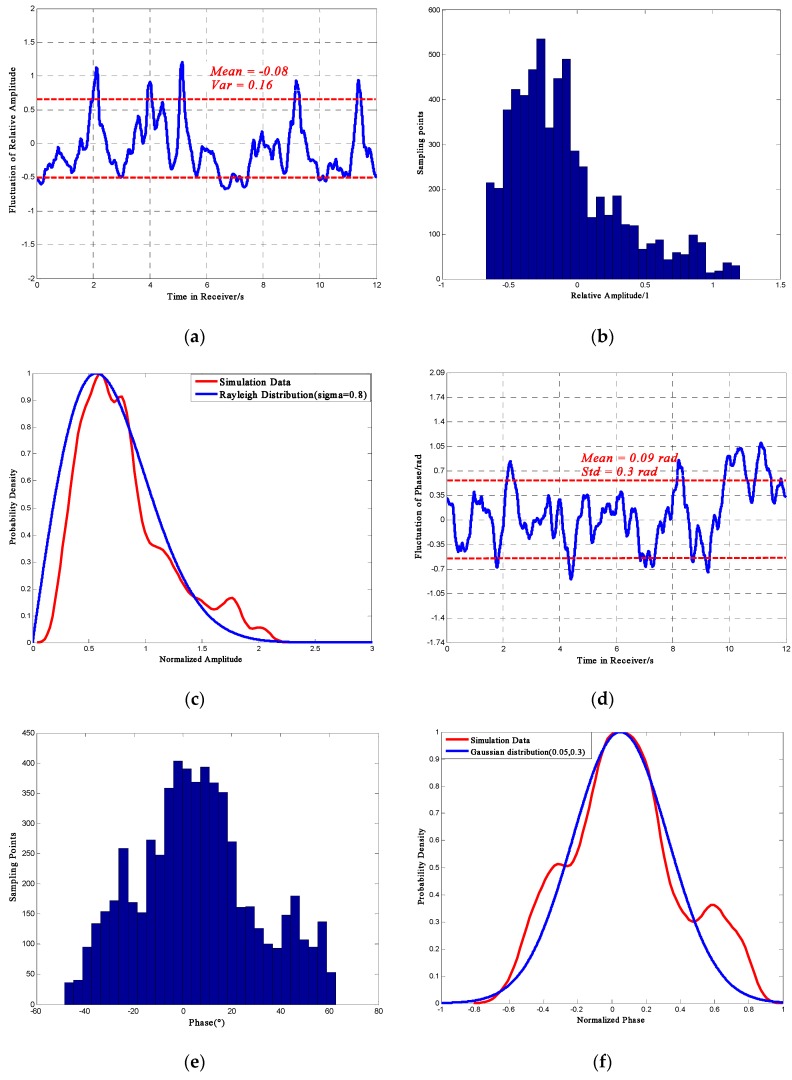
Phase and amplitude for *S*_4_ = 0.47. (**a**) Relative Amplitude Fluctuation; (**b**) Distribution of Amplitude Fluctuation; (**c**) Probability Density Curve; (**d**) Phase Fluctuation; (**e**) Distribution of Phase Fluctuation; (**f**) Probability Density Curve.

Figure 4 shows the distribution of phase and relative amplitude random fluctuation with *S*_4_ = 0.47. Note that the “Std” in the figures (and all the related figures in the paper) is standard deviation. Each result shown here is one sample profile, which is slightly different for every simulation. It should also be noted that the section in which the sampling point numbers are less than 150 should be rejected because the numbers are too small to be persuasive and representative. According to a 100 times simulations, the relative amplitude fluctuation could be −0.5 ± 0.05 to 0.4 ± 0.05, the phase deviation could be −0.52 ± 0.09 rad to 0.52 ± 0.09 rad. Furthermore, Figure 4c,f show the statistical probability density curves. The results show that under weak scintillation, phase fluctuation would largely follow a Gaussian distribution with *μ* = 0.05, *σ* = 0.3 and amplitude fluctuation would mostly follow a Rayleigh distribution with *σ* = 0.8.

**Figure 5:**
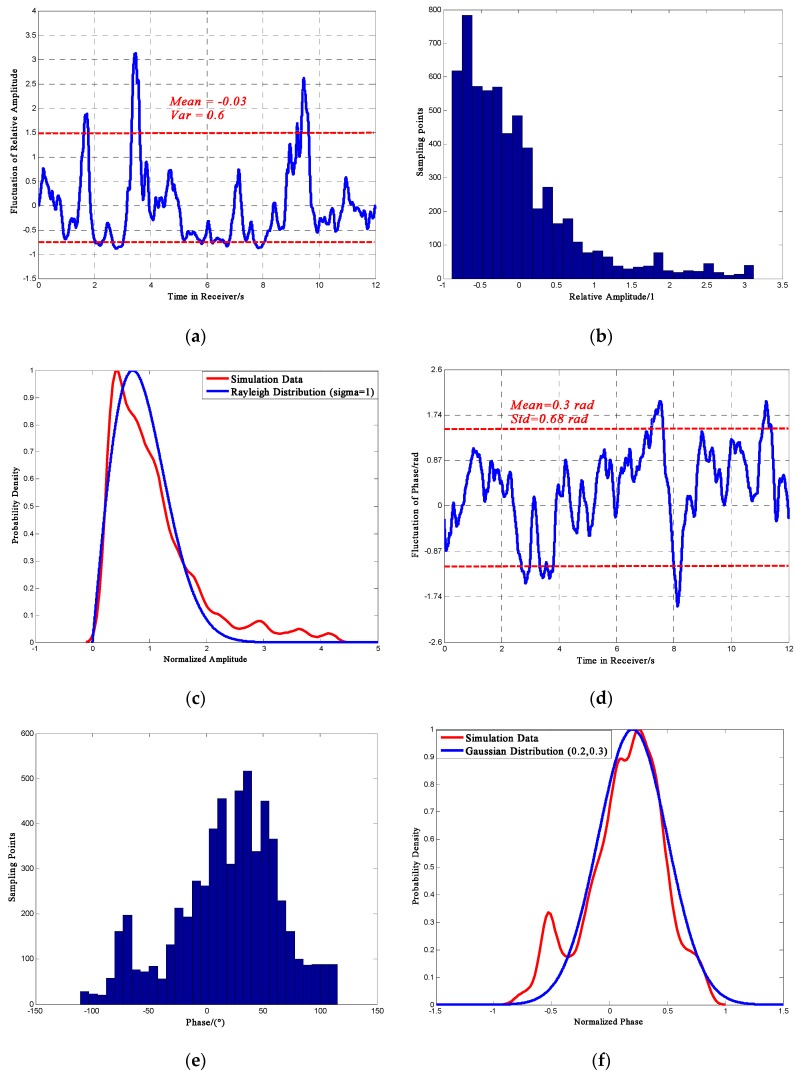
Phase and amplitude for *S*_4_ = 0.808 (**a**) Relative Amplitude Fluctuation; (**b**) Distribution of Amplitude Fluctuation; (**c**) Probability Density Curve; (**d**) Phase Fluctuation; (**e**) Distribution of Phase Fluctuation; (**f**) Probability Density Curve.

Figure 5 presents the distribution of phase and amplitude scintillation in strong scintillation with *S*_4_ = 0.808. Rejecting the section whose sample point number is below 150, the relative amplitude fluctuation could be −0.75 ± 0.05 to 0.8 ± 0.1, the phase deviation could be −1.13 ± 0.09 rad to 1.39 ± 0.09 rad.

Furthermore, it could be noticed that under strong scintillation, the fluctuation of phase agrees with a Gaussian distribution with *μ* = 0.2, *σ* = 0.3 and the amplitude fluctuation is getting closer to a Rayleigh distribution with *σ* = 0.8. In addition, the comparison of Figure 4 and Figure 5 shows that as the scintillation become stronger, the mean value of the relative amplitude differs slightly, but the variance is constantly increasing. At the same time for phase fluctuation, both of the mean value and variance would increase, which may result in receiver performance inaccuracy and instability.

## 4. Effect on the Acquisition Process for the BeiDou Receiver

According to BeiDou Interface Control Document (ICD) [25], the code rate of the BeiDou B1I (1561.098 MHz) signal is 2.046 Mbps with the code length 2046. As the chips of pseudo code are the smallest units of the BeiDou signal, this paper uses the BeiDou pseudo code to evaluate the influence of ionospheric scintillation on the BeiDou navigation signal. Besides, in order to analyze the influence only caused by ionospheric scintillations, this paper assumes that there exists no other factors that could cause errors for receiver.

**Figure 6:**
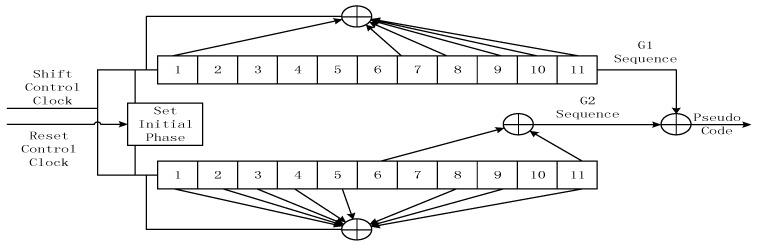
Sketch of BeiDou pseudo code generation.

The schematic of the generation of the BeiDou pseudo code (version 2.0) is shown in Figure 6.

**Figure 7:**
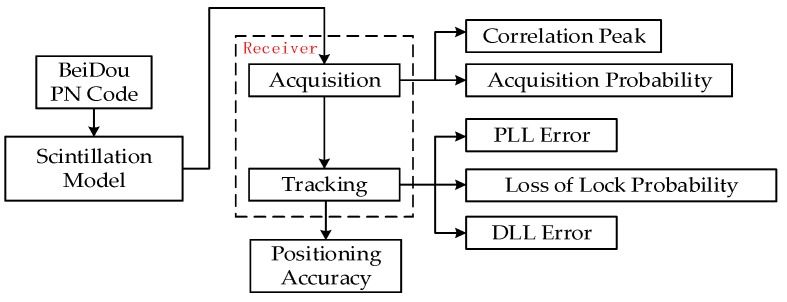
Analysis method.

The process of a received signal in a receiver mainly consists of two steps: acquisition and tracking, so this paper mainly analyzes the scintillation effects on these two aspects to evaluate the influence on receiver performance. Figure 7 shows the analysis method used in this paper.

For signal acquisition, the correlation peak and acquisition probability are two of the most important parameters to determine whether or not the signal could be captured by a receiver [26]. As the first step for a receiver to work, signal acquisition is related to the strong autocorrelation ability of pseudo code. The received pseudo code must match with the local one created by receiver itself. Once the correlation peak value is higher than detection threshold, the signal can be captured. For the signal detection process, the detection probability *P_d_* and false alarm probability *P_fa_* are determined as follows [26]:

(36) $$P_{d}=\int_{V_{t}}^{\infty }p_{s}(z)dz$$

(37) $$P_{fa}=\int_{V_{t}}^{\infty }p_{n}(z)dz$$

In Equations (36) and (37), *p_s_*(*z*) is probability density function (PDF) of the envelope with signal present, *p_n_*(*z*) is the envelope with signal absent, *V_t_* is the threshold. Assume that the envelope of signal is $\sqrt{I^{2}+Q^{2}}$, in which *I* and *Q* have a Gaussian distribution. Then *p_s_*(*z*) and *p_n_*(*z*) could be defined by:

(38) $$p_{s}(z)=\frac{z}{\sigma_{n}^{2}}e^{-(\frac{z^{2}}{2\sigma_{n}^{2}}+C/N)}I_{0}(\frac{z\sqrt{2C/N}}{\sigma_{n}})$$

(39) $$p_{n}(z)=\frac{z}{\sigma_{n}^{2}}e^{-(\frac{z^{2}}{2\sigma_{n}^{2}})}$$

In Equations (38) and (39), $\sigma_{n}^{2}$ is Root Mean Square (RMS) noise power, *C*/*N* is the pre-detection signal to noise ratio (SNR), *I*_0_(·) is a modified Bessel function with zero order. For a given false alarm *P_fa_*, the detection threshold is obtained as follows:

(40) $$V_{t}=\sigma_{n}^{2}\sqrt{-2\text{ln}P_{fa}}$$

Then the detection probability *P_d_* is:

(41) $$P_{d}=Q(\frac{p\cdot N\sqrt{2(C/N)}}{\sigma_{n}},\frac{V_{t}}{\sigma_{n}^{2}})$$

In Equations (41), *Q*(·) is the generalized Marcum-Q function, *N* is pseudo code length, *p* is the scintillation factor which is defined as the ratio of the cross-correlation peak and the pseudo code autocorrelation peak.

**Figure 8:**
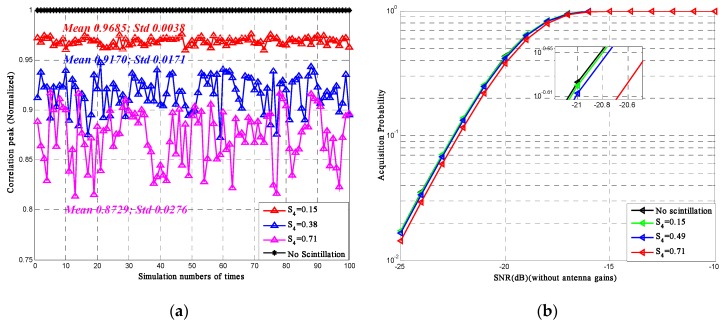
(**a**) Correlation peak of the BeiDou pseudo code (*N* = 2046); (**b**) Acquisition probability of the BeiDou signal (*N* = 2046).

According to Equations (40) and (41), for given *P_fa_* and $\sigma_{n}^{2}$, the acquisition probability of the BeiDou signal is obtained. Figure 8a shows the correlation peak of the BeiDou pseudo code under three levels of *S*_4_ for 100 simulations. The figure shows that when *S*_4_ is around 0.15, the average correlation peak could be degraded by 3%; when *S*_4_ is around 0.38, the degradation could be 8%; when *S*_4_ is around 0.71, the degradation could be up to 13%. The correlation variance also tends to become higher as the scintillation becomes higher indicating that the acquisition process would be less stable. Figure 8b shows the acquisition probability under different scintillation intensity scenarios resulting from the decrease of correlation peak. 

The simulation results shown above are under the assumption that there exist no fading during the signal propagation, which means the input signal power is equal to the received signal power. The curves indicate that the average correlation peak decreases as the scintillation intensity increases. Under the same SNR scope, the acquisition probability decreases as the scintillation intensity increases.

## 5. Effect on Tracking Process for BeiDou Receiver

Once the acquisition is achieved, the signal will be sent to the tracking loop in which the several kinds of information for positioning could be figured out. The tracking process mainly consists of the carrier tracking loop, including phase lock loop (PLL) and frequency lock loop (FLL), and code tracking loop (DLL). Since ionospheric scintillation will not influence the signal frequency, only PLL should be taken into consideration. The output of PLL is the carrier phase. If the error of carrier phase measurements is too high, the accuracy of carrier-phase-ranging will decrease and there is a high probability of the signal losing its lock. Even if the signal transmitted from a satellite is not lost, it can alter the position precision in DLL. One of the DLL functions is the measurement of the delay between the code carried by the signal and the receiver local code. This delay is an estimation of the time needed by a signal to reach the receiver. Then the receiver is able to compute the distance of the satellite. If the error of DLL is too high, it will have a great influence on pseudo-ranging, so considering all the factors mentioned, this paper uses the correlation peak, acquisition probability, PLL error, loss of lock probability and DLL error to evaluate the influence of scintillation on receiver performance [27].

### 5.1. Determining PLL Variance for the BeiDou B1I Signal

After the signal acquisition, the carrier frequency is evaluated, then the receiver performs a tracking process with carrier tracking loop (PLL and FLL) and PN code tracking loop (DLL). As the ionospheric scintillations won’t have an effect on signal frequency, this paper would mainly discuss the influence on PLL.

For the carrier tracking loop, once the receiver fails to track the carrier phase, the signal is lost. Loss of lock is related to the tracking error variance at the output of the PLL. This variance could be expressed as follows [7]:

(42) $$\sigma_{\Phi }^{2}=\sigma_{\Phi S}^{2}+\sigma_{\Phi T}^{2}+\sigma_{\Phi osc}^{2}$$

In Equation (42), *σ*_Φ*S*_ is the phase scintillation, *σ*_Φ*T*_ is the thermal noise, *σ*_Φ*osc*_ is the receiver oscillator noise (usually 0.122 rad). The phase variance at the output of the PLL is:

(43) $$\sigma_{\Phi S}^{2}=\int_{-\infty }^{+\infty }{\left|1-H(f)\right|}^{2}\Phi_{\xi }(\vec{f})df$$

In Equation (43), $\Phi_{\xi }\left(\vec{f}\right)$, given in Equation (12), is the PSD of phase scintillation provided by the MPS model. ${\left|1-H\left(f\right)\right|}^{2}$ is the closed loop transfer function of the PLL. According to [28], the thermal noise tracking error could be characterized by *S*_4_, which is given by:

(44) $$\sigma_{\Phi T}^{2}=\frac{B_{n}(1+\frac{1}{2t_{0}(C/N_{0})(1-2S_{4}^{2})})}{\left(C/N_{0})(1-2S_{4}^{2}\right)}$$

In Equation (44), *C*/*N*_0_ is the SNR which contains the antenna gains about 40 dB, *B_n_* is the third-order PLL one-sided bandwidth equal to 10 Hz for the BeiDou B1I signal, *t*_0_ is the pre-detection integration time, equal to 0.02 s for the BeiDou signal. Based on Equations (43) and (44), the PLL tracking error variance can be computed.

**Figure 9:**
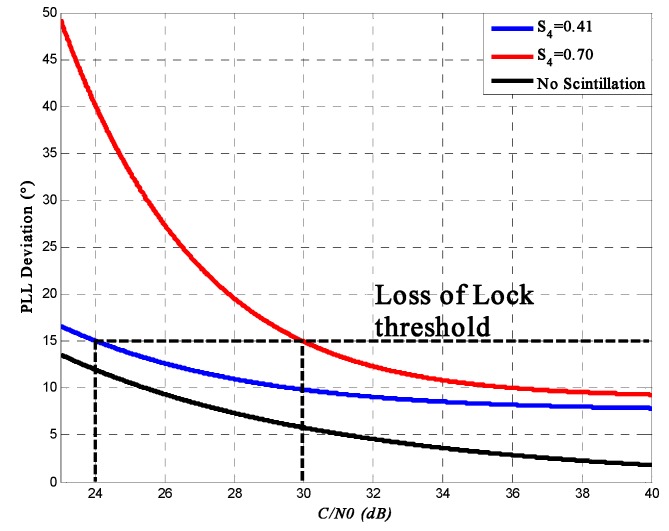
PLL standard deviation.

Figure 9 shows the variance with *S*_4_ = 0.70 and *S*_4_ = 0.41. By the observation of 100-repetition simulation data, we find that the value of the phase scintillation is far smaller than that of the thermal noise, and the thermal noise appears to be the decisive contributor to the PLL tracking error. The figure shows that as the signal SNR increases, the phase error of the PLL output decreases and that the influence of intense scintillation on PLL is much higher than that of weak scintillation when the signal SNR is relative lower. For PLL, there always exits a tracking threshold: once the signal *C*/*N*_0_ is lower than this threshold, the receiver can’t continue tracking such a weak signal stably, which is also called the loss of lock. According to [26], one method for establishing this threshold is that the triple of the PLL variance can’t be more than a quarter of the scope of the phase discriminator:

(45) $$3\times \sigma_{PLL}\leq \frac{1}{4}\times 180{}^{\circ}$$

Figure 9 also shows this threshold. From the figure, loss of lock is highly probable for values above the 15° threshold, therefore a receiver can’t tolerate scintillation if the *C*/*N*_0_ (in dB) is below a maximum, which is 24 dB for *S*_4_ = 0.41 and 29.5 dB for *S*_4_ = 0.70.

On the other hand, one of the outputs of PLL is the measurement of carrier phase, which is an essential parameter for carrier phase ranging. Carrier phase ranging uses the phase difference between the received signal and the initial signal to calculate the distance from the satellite to the receiver. Figure 9 shows that if the signal *C*/*N*_0_ is 26 dB, the phase error under *S*_4_ = 0.70 and *S*_4_ = 0 could be 27° and 8°. Then for the BeiDou B1I signal (*λ* = 19.2 cm), the error of carrier phase ranging caused by scintillations *S*_4_ = 0.70 could be $\frac{2\times \left(27{}^{\circ}-8{}^{\circ}\right)}{360{}^{\circ}}\cdot \lambda$ = 2.02 cm, which would have a relatively severe effect on the precise point position in the carrier phase ranging method.

All the calculations above ignore the signal fading, but when it comes to calculating the loss of lock probability, it is necessary to take fading into account, which could consequently lead to a degradation of SNR at the receiver. According to [7], Equation (44) could be changed into:

(46) $$\sigma_{\Phi T}^{2}=\frac{B_{n}}{(C/N_{0})F}(1+\frac{1}{2t_{0}(C/N_{0})F})$$

In Equation (46), *F* is the amplitude scintillation intensity randomly distributed with parameter $S_{4}$. Based on 100 times of simulation, the distribution of normalized amplitude scintillation under different $S_{4}$ corresponds to a Nakagami-m distribution, so the probability density function of *F* could be expressed in terms of a Nakagami-m fading model:

(47) $$P(z)=\frac{2m^{m}z^{2m-1}}{\Omega^{m}\Gamma (m)}\text{exp}(-\frac{mz^{2}}{\Omega })$$

In Equation (47), Ω is a scale factor, equal to the mean power of the normalized intensity, $m=1/S_{4}^{2}$ is the shape factor, Γ(·) is a gamma function. For a given *C*/*N*_0_, *F* must be below a threshold to meet the requirement that *σ*_Φ_ be above 15°. Then the probability of event “F < threshold” can be evaluated, which represents the loss of lock probability.

**Figure 10:**
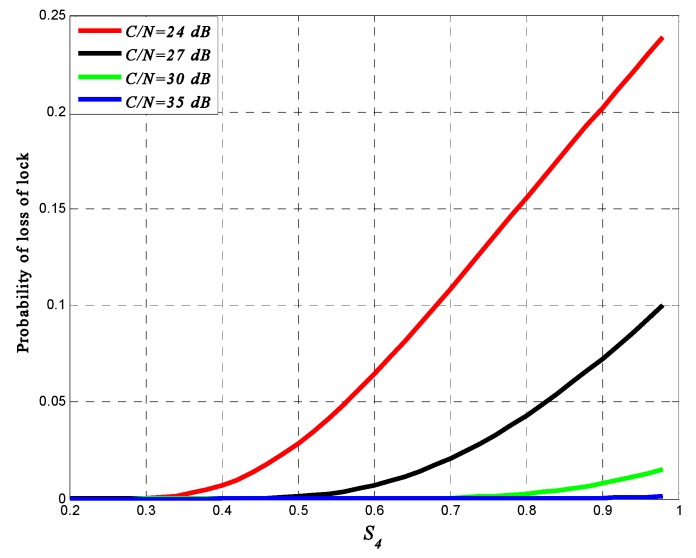
Probability of loss of lock.

Figure 10 presents this probability at different given values of the *C*/*N*_0_ for the BeiDou signal. From the figure, we can see that under the same *C*/*N*_0_, the loss of lock probability increases with the increase of scintillation intensity and the probability increases slower as the *C*/*N*_0_ decreases. Under high scintillation intensity and low *C*/*N*_0_ level, the loss of lock probability could be 0.11 to 0.26. In some extreme cases (e.g., *S*_4_ > 0.9, *C*/*N*_0_ < 20 dB), the loss of lock probability could reach 0.3 or even more. It is also can noticed that, under high scintillation intensity, links with high SNR are quite robust. On the contrary, links with low values of SNR are likely to be lost.

### 5.2. Determining DLL Variance for BeiDou B1I Signal

For the code tracking loop, if carrier tracking is not lost due to amplitude or phase scintillation, the problem becomes by what degree code tracking errors increase. Phase scintillations are ignored in that they have been filtered in PLL and thus have little effect on code tracking errors. We only need to be concerned with tracking code errors caused by thermal noise and amplitude scintillations. 

In the presence of scintillation and thermal noise jitter variance, the tracking variance for a DLL in C/A code chips squared could be expressed as:

(48) $${\sigma_{T}}^{2}=\frac{B_{n}d[1+\frac{1}{\eta (C/N_{0})(1-2S_{4}^{2})}]}{2(C/N_{0})(1-S_{4}^{2})}$$

In Equation (48), *B_n_* is the one-sided noise bandwidth, and *d* is the correlator spacing in C/A chips. Such expression is derived in a manner completely analogous to the derivation of the thermal noise jitter variance for a PLL.

Then the standard deviation of DLL tracking jitter in meters is:

(49) $$\sigma_{\hat{T}}=W_{B1I}\sigma_{T}$$

**Figure 11:**
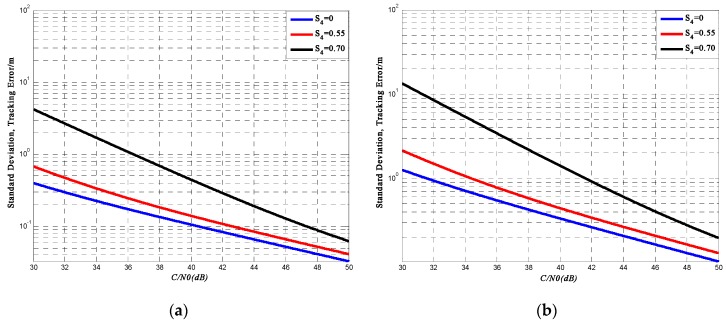
(**a**) BeiDou PN code tracking error (*d* = 1); (**b**) BeiDou PN code tracking error (*d* = 0.1).

In Equation (49), *W_B_*_1*I*_ is the chip length for the BeiDou B1I signal. Figure 11a,b show respectively the results of BeiDou B1I code tracking for *d* = 1 and *d* = 0.1 for the BeiDou receiver. Displayed are the values of $\sigma_{\hat{T}}$ with respect to *C*/*N**_0_*** for different levels of ionospheric scintillation. For a typical BeiDou receiver, *B_n_* is equal to 0.1 Hz, *η* is equal to 0.02 s, *W*_*B*1*I*_ is 146.526 m. Simulation curves show that under high scintillation intensity, DLL tracking errors could be more than 10 m when *d* = 1 and close to 5 m when *d* = 0.1. 

The calculation of DLL error ignores the influence of phase scintillation on the tracking process, but in order to accurately analyze the influence on high precision positioning, which is closely related to the pseudo-range accuracy, we should take it into consideration.

**Figure 12:**
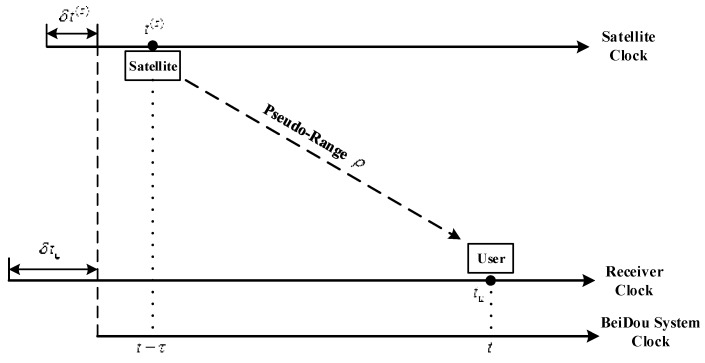
Pseudo ranging.

In order to obtain the accurate location of a user, the receiver needs to measure the accurate distance from the receiver to the satellite, which is the so-called pseudo-range. As is shown in Figure 12, a satellite sends out the navigation signal at *t*^(*s*)^ and get received by user receiver at *t_u_*.

Generally, the clock in a satellite and receiver do not synchronize with the system clock, so there always exists a clock error. In correspondence with the BeiDou system clock *t*, the satellite clock and receiver clock can be written as:

(50) $$t_{u}(t)=t+\delta t_{u}(t)$$

(51) $$t^{\left(s\right)}(t)=t+\delta t^{\left(s\right)}(t)$$

Assume that the actual signal propagation time is *τ*, then according to Equation (51), at moment (*t* − *τ*):

(52) $$t^{\left(s\right)}(t-\tau )=t-\tau +\delta t^{\left(s\right)}(t-\tau )$$

Then pseudo-range ρ(t) is defined as:

(53) $$\rho (t)=c(t_{u}(t)-t^{\left(s\right)}(t-\tau ))=c\tau +c(\delta t_{u}(t)-\delta t^{\left(s\right)}(t-\tau ))$$

In Equation (53), *c* is the speed of light. Signal propagation time *τ* contains the time delay caused by the background ionosphere and troposphere. In order to study the influence only caused by ionospheric scintillation, this paper does not take into consideration the influence of background ionosphere and troposphere. 

Since *t_u_*(*t*) could be directly obtained from the receiver clock, we only need to figure out *t*^(*s*)^(*t*) to calculate the pseudo-range ρ(t). Actually, the direct measurement from the receiver is not ρ(t) or *t*^(*s*)^(*t−τ*) but the code phase (CP), which is obtained from the C/A code correlator in the receiver. In the presence of scintillation, *t*^(*s*)^(*t*) could be figured out (for the BeiDou signal) by:

(54) $$t^{\left(s\right)}=TOW+(30w+b)\times t_{bit}+(d+\frac{CP+\Delta CP}{N})\times t_{chip}$$

**Figure 13:**
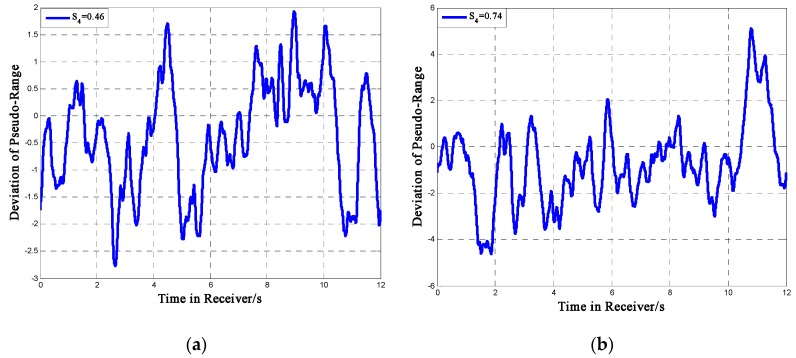
Deviation of Pseudo-Range (**a**) *S*_4_ = 0.46; (**b**) *S*_4_ = 0.7.

In Equation (54), *TOW* is the time of week, *w* and *b* are the number of words and bits of navigation message the receiver has received by the current sampling moment, *t_bit_* and *t_chip_* are the time periods of one single bit and chip, Δ*CP* is the code phase jitter caused by ionospheric scintillation, *N* is the number of chips in one period of PN code, which is 2046 for the BeiDou signal. Then we could obtain an expression of ρ(t) as a function of Δ*CP*. Figure 13 shows the deviation of pseudo-range in 12 s, which is the period of two sub-frames of the BeiDou signal.

**Figure 14:**
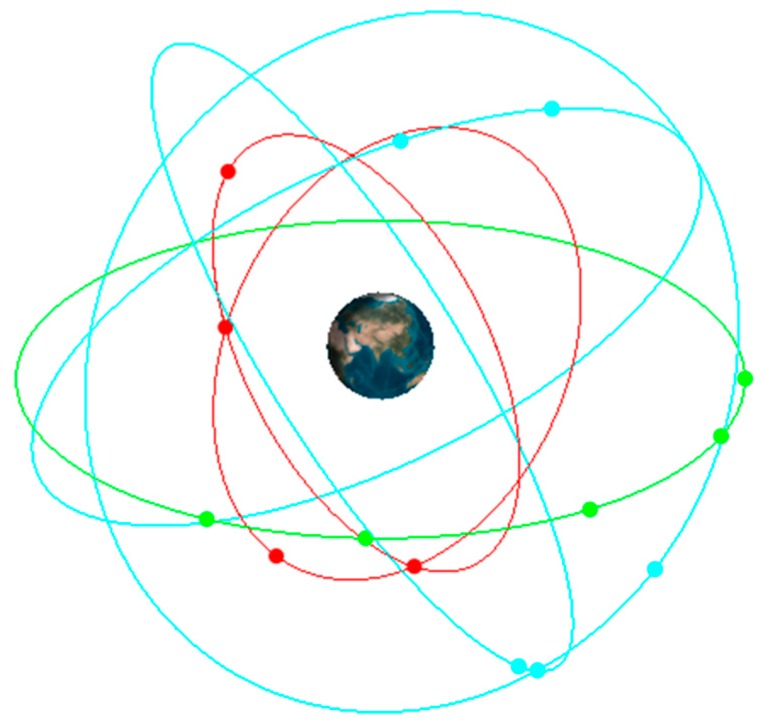
BeiDou satellites.

From the figure we can see that under moderate scintillation, the deviation could be within ±2 m; when the intensity becomes higher, the deviation of pseudo-range caused by ionospheric scintillation could be more than 4 m under high scintillation intensity. The pseudo-range error could directly affect the receiver positioning accuracy. Considering that this paper mainly focuses on some low-latitude areas, we choose Sanya as an example. Sanya (N18°E109°) is a city located in Southern China and the receiver is assumed to be in the center of the city. In this case, only the impact of ionospheric scintillation is taken into consideration. The 14 BeiDou satellites are built in Satellite Tool Kit (STK) as shown in Figure 14, where the red point is a MEO satellite; the green point is a GEO satellite and the blue one is an IGSO satellite. The simulation period is from 07:00 a.m. to 10:00 a.m. The positioning accuracy could be evaluated by the deviation between the actual value (N18°E109°) and the calculation results. More details of the computational setup are shown in Table 2.

**Figure 15:**
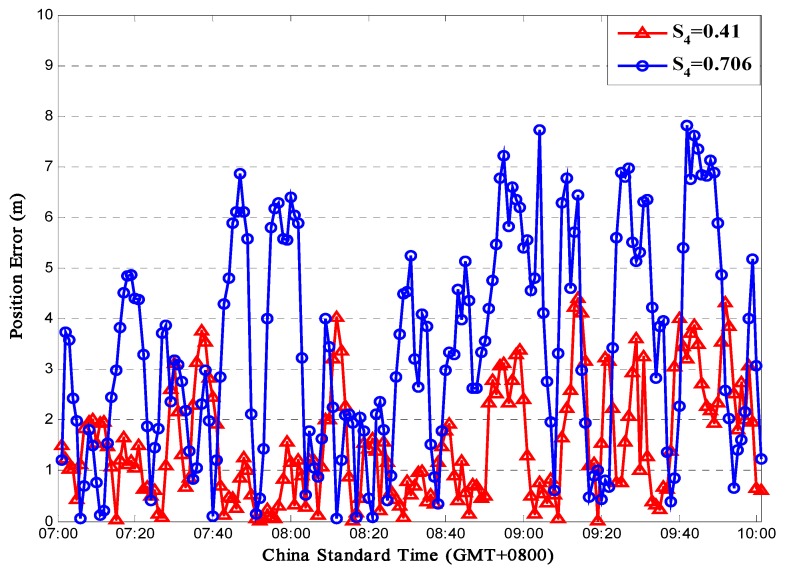
Positioning Accuracy for Sanya (N18°E109°).

Figure 15 presents the positioning error under two scintillation levels. According to the simulation, when *S*_4_ = 0.41, the mean error is 1.52 m and the standard deviation is 1.1 m; when *S*_4_ = 0.706, the mean error would be 3.4 m and the standard deviation would be 2.13 m. Under strong scintillation, sometimes the error could be more than 6 m, which could have a huge impact on some positioning-based applications like vehicle navigation systems. Besides, according to the simulation data, the simulation time for each position process is about 1.5 s. Since Matlab needs to do real-time communication with the STK software to obtain data, the actual process time in a receiver would be much shorter. 

## 6. Discussion and Conclusions

Firstly, by using a multiple phase screen model, this paper presents the influence of ionospheric scintillation on satellite signals for equatorial and mid-latitude areas. Sample profiles show that when the scintillation intensity increases, the random phase and amplitude fluctuation become stronger. When the scintillation intensity $S_{4}$ is close to 0.7 or even higher, the phase fluctuation could be −1.05 ± 0.09 rad to 0.87 ± 0.09 rad and the relative amplitude fluctuation could be up to −10 dB to 8 dB. Then by using the BeiDou pseudo code as the input signal, this paper evaluates the impact of ionospheric scintillation on two receiver processes: acquisition and tracking.

For the acquisition process, this paper focuses on the acquisition probability and correlation peak. The results show that the acquisition probability decreases as the scintillation intensity increases. Under high scintillation levels, the average correlation peak could be 13% lower. Such a decrease could thus result in the degradation of the acquisition performance to some extent and this degradation increases as the signal SNR decreases. What’s more, the results also show that the fluctuation of the correlation peak become more intense with stronger scintillation. Such a phenomenon indicates that for a given detection threshold, the stability and robustness of the acquisition process would be degraded under intense scintillations. For some cases in which $S_{4}$ is higher than 0.7, the degradation of acquisition performance could be more than 13%. 

For the tracking process, this paper mainly focuses on PLL variance, loss of lock probability, and DLL variance and thereby analyzes the influence on ranging accuracy. PLL simulation results show that as the signal SNR increases, the phase error of the PLL output decreases. Under the same SNR, the phase error with high scintillation intensity is higher than that with low scintillation intensity. It also shows that when the signal SNR is relatively lower, the influence of intense scintillation on PLL is much higher than that of weak scintillation. Moreover, the output of PLL is used to calculate the carrier-phase range measurement. The error caused by intense scintillation could affect the position accuracy by about 2–3 cm, which is a relatively high error for a carrier-phase ranging method. Based on the PLL variance, loss of lock probability is presented. Under the same SNR, the loss of lock probability increases with the increase of scintillation intensity. Under high scintillation intensity and low SNR level, tracking loop is much easier to get lost. For a receiver, the output of DLL is the code phase, which is used to compute the pseudo-range measurement. Simulation results show that under high scintillation intensity, the pseudo-ranging error could be more than 10 m when the correlator spacing *d* = 1. The calculation of DLL variance only takes amplitude scintillation into consideration and is shown as a function of SNR, so in order to analyze the results more accurately, this paper also presents the effects of phase scintillation on position accuracy in the time-domain. Simulation results also show that the positioning error caused by ionospheric scintillation could be more than 6 m under strong scintillation, which could have a huge harmful impact on some positioning-based applications.

As a whole, for BeiDou receivers, ionospheric scintillations could lower the final position accuracy. In some extreme cases, they could cause acquisition and tracking failure. Considering the geographical location of Southern China, where the ionospheric scintillation is more severe and occurs more frequently, the study of the impact on BeiDou performance is significant. Additionally, this research could provide some help for the design of high-accuracy BeiDou receivers in which the impact of ionospheric scintillation needs to be compensated.

## Figures and Tables

**Table 1:** Simulation parameters.

Parameter	Value
Carrier Frequency (MHz)	1561.098
Ionosphere Altitude (km)	200–400
Power Law Index *p*	4
False Alarm Probability	10^−6^
Classical Electron Radius (m)	2.818 × 10^−15^
Code Rate of BeiDouB1I Signal (Mbps)	2.046
Sample Points	6000
Sampling Frequency	500
Outer Scale (m)	5000
Inner Scale (m)	15
Distance Between two Adjacent Screens (m)	5000
Average Electron Density	9 × 10^11^
Computer Language	Matlab

**Table 2:** Computational set up for positioning example.

Location of Receiver	N18°, E109°
Date (GMT + 0800)	21 March 2016
Time period (GMT + 0800)	07:00 a.m. to 10:00 a.m.
Positioning Satellites	Three MEO and one IGSO
Time Sampling Interval	60 s
Positioning Method	Single Point Positioning
Satellite Clock Error	0
Coordinate System	J2000
Total Sample Points	181
